# Brief mindfulness training alters causal brain connections in mTBI

**DOI:** 10.1186/1471-2202-16-S1-P247

**Published:** 2015-12-18

**Authors:** Rongxiang Tang, Yi-Yuan Tang

**Affiliations:** 1 Department of Psychology, The University of Texas at Austin, Austin, TX 78712, USA; 2 Department of Psychological Sciences, Texas Tech University, Lubbock, TX 79409, USA

## 

Traumatic brain injury (TBI) is a significant cause of disability in the United States and the mild TBI (mTBI) is the most prevalent. Previous research indicates the positive effect of mindfulness training on symptoms of chronic mTBI such as cognitive functioning and emotion [1]. However it remains unclear which brain regions play a crucial role in mTBI recovery and the causal relationship among these regions. Here we apply a dynamic causal modeling (DCM) in resting state fMRI [2] to demonstrate the causal relationships among the core regions involved in mTBI.

Fourteen veteran students with mTBI were recruited through campus advertisements. We used 2 weeks of integrative body-mind training as mindfulness intervention, previously reported in our series of randomized studies [3,4]. All data were collected using a 3-Telsa Siemens Skyra scanner. Functional data were processed using the Data Processing Assistant for Resting-State fMRI, which is based on SPM and Resting-State fMRI Data Analysis Toolkit. Based on literature, we specified four regions of interest within default mode network (DMN) - medial prefrontal cortex (mPFC), posterior cingulate cortex (PCC), and bilateral inferior parietal lobule (Left IPL and Right IPL), same regions and coordinates as in previous sDCM studies [2,5]. Based on SPM12, we estimated and specified the DCM for each subject and later compared the differences of effective connectivity before and after mindfulness. Our results suggest the different causal relationships following mindfulness training. Specifically, after training there was a significant decrease in the strength of excitatory input from mPFC to PCC, and a significant increase in the strength of inhibitory input from mPFC to LIPL (all p<0.05).

## Conclusions

Resting DCM can differentiate the causal brain connections before and after mindfulness training, and provide insight into the brain mechanisms of altered DMN dynamics underlying mTBI recovery, suggesting the changes in information flow in these distributed systems involved in mTBI intervention.
